# Enlargement of Gold Nanoparticles for Sensitive Immunochromatographic Diagnostics of Potato Brown Rot

**DOI:** 10.3390/s19010153

**Published:** 2019-01-04

**Authors:** Shyatesa C. Razo, Natalia A. Panferova, Vasily G. Panferov, Irina V. Safenkova, Natalia V. Drenova, Yuri A. Varitsev, Anatoly V. Zherdev, Elena N. Pakina, Boris B. Dzantiev

**Affiliations:** 1 A.N. Bach Institute of Biochemistry, Research Centre of Biotechnology of the Russian Academy of Sciences, Leninsky Prospect 33, 119071 Moscow, Russia; kish218@gmail.com (S.C.R.); nemchenko.na@yandex.ru (N.A.P.); panferov-vg@mail.ru (V.G.P.); saf-iri@yandex.ru (I.V.S.); zherdev@inbi.ras.ru (A.V.Z.); 2 Agricultural-Technological Institute, RUDN University, Miklukho-Maklaya Street 8/2, 117198 Moscow, Russia; e-pakina@yandex.ru; 3 All-Russian Plant Quarantine Centre, Pogranichnaya Street 32, Bykovo-2, Moscow Region, 140150 Moscow, Russia; drenova@mail.ru; 4 A.G. Lorch All-Russian Potato Research Institute, Lorch Street 23, Kraskovo, Moscow Region, 140051 Moscow, Russia; varyuriy@yandex.ru

**Keywords:** gold nanoparticles, gold particle growth, immunochromatographic diagnostics, lateral flow immunoassay, test strips, potato brown rot, *Ralstonia solanacearum*, increase of sensitivity

## Abstract

Lateral flow immunoassay (LFIA) is a convenient tool for rapid field-based control of various bacterial targets. However, for many applications, the detection limits obtained by LFIA are not sufficient. In this paper, we propose enlarging gold nanoparticles’ (GNPs) size to develop a sensitive lateral flow immunoassay to detect *Ralstonia solanacearum*. This bacterium is a quarantine organism that causes potato brown rot. We fabricated lateral flow test strips using gold nanoparticles (17.4 ± 1.0 nm) as a label and their conjugates with antibodies specific to *R.* *solanacearum*. We proposed a signal enhancement in the test strips’ test zone due to the tetrachloroauric (III) anion reduction on the GNP surface, and the increase in size of the gold nanoparticles on the test strips was approximately up to 100 nm, as confirmed by scanning electron microscopy. Overall, the gold enhancement approach decreased the detection limit of *R. solanacearum* by 33 times, to as low as 3 × 10^4^ cells∙mL^–1^ in the potato tuber extract. The achieved detection limit allows the diagnosis of latent infection in potato tubers. The developed approach based on gold enhancement does not complicate analyses and requires only 3 min. The developed assay together with the sample preparation and gold enlargement requires 15 min. Thus, the developed approach is promising for the development of lateral flow test strips and their subsequent introduction into diagnostic practice.

## 1. Introduction

Lateral flow immunoassay (LFIA) is a convenient tool for rapid field-based control of important analytes for the purposes of medicine, veterinary medicine, food quality, and environmental safety [1,2,3,4]. LFIA combines the principle of chromatographic separation of reagents and highly specific immunochemical interactions [5]. However, the high limit of detection (LOD) is a major drawback for LFIA. Reducing the LOD is an important task in developing LFIA test systems.

Recently, many studies have suggested a variety of approaches to decrease the LOD of LFIA; see a review for details [6,7,8]. However, none of these approaches is universal [9]. At the same time, the approaches that are promising and deserve special attention are based on increasing the size of the label immediately after the formation of a specific immune complex in the test zone. The realization of such signal amplification should start with the use of reagents specifically bound in the test zone: nanoparticles as seeds for growth [10], nanozymes with catalytic properties [11], or conjugated enzymes [12,13]. Below are examples of the use of gold nanoparticles (GNPs) as labels that combine catalytic properties with seed growth.

GNPs exhibit catalytic properties and serve as seeds for growth in the reduction reaction of silver salts in the presence of various reducing agents (hydroquinone, 4-(methylamino) phenol sulfate) [14,15,16]. This approach can decrease the LOD of LFIA for various antigens: fumonisin B1 and deoxynivalenol [17], ochratoxin A [18], cadmium [19], potato virus X [20], potato leafroll virus [21], *Helicobacter pylori* [22], and others. The silver enhancement method is characterized by a high increase in the color intensity [17,18,23], and it takes only a little time (up to 10 min) and is universal for LFIAs based on GNP conjugates [15]. However, the disadvantages of the method are the low stability of the initial reagents [24] and the need to remove chloride and phosphate ions (matrix components) because of the formation of slightly soluble silver salts, which leads to a significant decrease in the efficiency of silver amplification [22].

A second promising approach is the use of the GNP enlargement in the reduction reaction of tetrachloroauric (III) anions (gold enhancement method) [25,26]. The GNPs can act as a seed for growth and increase in size themselves. This approach was used to reduce the detection limit of pesticides in multiplex LFIA [27], potato virus X [28], and *Salmonella enteritidis* [29]. Moreover, Dias et al. illustrated that the gold enhancement method can be used to lower the LOD of bioanalytical systems with various markers—silver, magnetic particles, and particles of silicon dioxide [10]. After optimizing the concentrations of reagents and the amplification time, the detection limit was reduced by 100 times [10]. Gold enhancement has several advantages compared to silver enhancement: the possibility of amplification in the presence of chloride and phosphate ions without additional washes [30], the speed of amplification [29,31], and the high stability of the reagents for amplification [31].

Based on the abovementioned characteristics, we chose the gold enhancement method to reduce the detection limit of LFIA for *Ralstonia solanacearum*, known as a quarantine bacterium and the causal organism for brown rot in potatoes [32]. *R. solanacearum* is a pathogenic bacterium that causes a virulent vascular wilt disease in many plant species [33]. It ranks 2nd among the top 10 pathogenic bacteria in plant pathology [34]. Yield loss varies according to the host and cultivars; for example, it ranges over 33–90% for potatoes, 0–91% for tomatoes, 10–30% for tobacco, 80–100% for bananas, and up to 20% for peanuts [35]. The disease can cause global damage of over $950 million each year in approximately 80 countries worldwide [36]. Thus, highly sensitive and quick methods are needed for the detection of the pathogen in plant material. Many reports have utilized polymerase chain reaction (PCR)-based assays for *R. solanacearum* detection [37,38,39,40]. However, these sensitive instrumental methods cannot meet the need for sensitive and rapid field methods. Previously, we developed an LFIA with silver enhancement for the detection of *R. solanacearum*, which improved the sensitivity by 10 times [41]. However, taking into account the advantages of gold enhancement noted above, we expect that gold enhancement will be more suitable for solving the practical task of diagnosing potato brown rot.

## 2. Materials and Methods

### 2.1. Materials

Strain NCPPB No. 2316 of *R. solanacearum* was acquired from the National Collection of Plant Pathogenic Bacteria (London, UK); strains of other widespread potato pathogens (*Clavibacter michiganensis* subsp. *sepedonicus*, *Pectobacterium carotovorum, Dickeya* sp.), strains isolated from other *Solanaceae* plants (*C. michiganensis* subsp. *michiganensis*, *Artrobacter castelli*), and *Pseudomonas syringae* as a species from a close genus were acquired from the collection of the All-Russian Plant Quarantine Centre (Moscow region, Russia). Polyclonal antibodies specific to *R. solanacearum* were obtained from the A.G. Lorch All-Russian Potato Research Institute (Moscow region, Russia). We also used protein A derived from *Staphylococcus aureus* (Imtek, Moscow, Russia), tetrachloroauric (III) acid trihydrate, 2-aminoethanol hydrochloride, bovine serum albumin (BSA), and sodium azide (Sigma Aldrich, St. Louis, MO, USA). All the salts, acids, and solvents were of analytical reagent or chemical reagent grade.

The LFIA test strips were assembled using membranes manufactured by Advanced Microdevices (Ambala Cantt, Haryana, India): working nitrocellulose membranes CNPC-12μ, a glass fiber membrane for applying a conjugate PT-R5, a membrane for applying the sample GFB-R4, and the final adsorbing membrane AP045. To separate the protein molecules and their conjugates from low-molecular-weight compounds, a dialysis bag (10 × 6 mm) from Sigma-Aldrich (St. Louis, MO, USA) was used. All solutions were prepared using deionized water from a MilliQ (Millipore, Burlington, MA, USA) filter.

### 2.2. Synthesis of Gold Nanoparticles

GNPs were obtained by reduction of HAuCl_4_ with sodium citrate (the Frens method) [42]. A quantity of 2 mL of HAuCl_4_ was added to prefiltered (0.22 μm filter) deionized water (190 mL) and brought to a boil. Then, 8 mL of 1% sodium citrate was added to the solution, and the solution was boiled for 35 min with vigorous stirring using a reflux condenser. The synthesized GNPs were stored at 4–6 °C.

### 2.3. Synthesis and Characteristic of Gold Nanoparticle–Antibody Conjugates

Physical adsorption was used for the synthesis of GNP–antibody conjugates [43]. To determine the number of biomolecules for the synthesis of conjugates, the minimum stabilizing concentration was determined using the flocculation curve. Antibodies were dialyzed against 10 mM Tris buffer (pH 9.0). To obtain the flocculation curve, 200 µL of GNPs was incubated with antibodies (final concentration was varied from 0 to 18 μg∙mL^−1^) for 10 min. Next, NaCl to the final concentration of 1% was added. The optical density at 580 nm was measured using an EnSpire plate reader (Perkin Elmer, MA, USA). For the synthesis of conjugates, we used the concentration of antibodies, exceeding by 10–15% the point at which the curve reached the plateau. Solutions of GNPs (10 mL, A_520_ = 1) and antibody (at the optimal concentration that was selected according to the flocculation curve) were mixed and incubated for 60 min with constant stirring. Next, bovine serum albumin (BSA) was added to reach a final concentration of 0.25%, and it was incubated for a further 15 min. The GNP–antibody conjugate and unbound antibodies were separated by centrifugation (18,000 *g*, 30 min) and re-suspended in a conjugate buffer (20 mM Tris-HCl, pH 7.5, containing 1% BSA, 1% sucrose, 0.1% sodium azide, and 0.25% Tween-20). Finally, the optical density at a wavelength of 520 nm (A_520_) was measured using a Libra S80 spectrophotometer (Biochrom, Cambridge, United Kingdom).

### 2.4. Transmission Electron Microscopy (TEM)

All TEM experiments were performed using a JEM-1400 microscope (Jeol, Tokyo, Japan) operated at 80 kV. The GNPs (10 μL) were adsorbed on the surface of a grid for 15 min, excess liquid was removed, and the grid was dried at room temperature. Digital images were analyzed using the Image Tool program (UTHSCSA, San Antonio, TX, USA).

### 2.5. Atomic Force Microscopy (AFM)

Samples of *R. solanacearum* (10 μL, 10^7^ cells·mL^−1^) in 0.05 M potassium phosphate buffer (pH 7.4, with 0.1 M NaCl (PBS)) and samples of GNPs and GNPs after gold enhancement (A_520_ = 1) were applied to the surfaces of freshly cleaved mica by dipping and incubated for 10 min. Then, the excess liquid was removed by filter paper. The AFM experiments were performed using a SmartSPM-1000 atomic force microscope (AIST-NT, Moscow region, Russia) in a tapping mode using fpN01HAR cantilevers (Nanotuning, Moscow region, Russia). The root mean square (RMS) was determined as the standard deviation of the z-values around the nanoparticle surface. The results were analyzed using Gwyddion v. 2.42 (Czech Metrology Institute, Brno, Czech Republic).

### 2.6. Dynamic Light Scattering (DLS)

Measurements of the hydrodynamic size of GNPs and GNP–antibody conjugates were performed using a Zetasizer Nano (Malvern Panalytical, Malvern, UK). The scattering angle was 173°. The temperature was stabilized at 25 °C. All measurements were repeated at least 100 times. Malvern Software v. 7.11 was used for data analysis.

### 2.7. Preparation of LFIA Test Strips

To dispense the reagents in the test and control zones on the nitrocellulose membranes in the form of lines, we used an IsoFlow dispenser (Imagene Technology, Hanover, NH, USA). Antibodies were applied in the amount of 0.15 μL·mm^−1^ at concentrations of 1.5 mg·mL^−1^ (specific polyclonal antibodies in the test zone) and 0.4 mg mL^−1^ (protein A in the control zone). The GNP–antibody conjugate was absorbed on a glass fiber membrane (width 5 mm) in the amount of 16·μL mm^−1^. The membranes were dried at 37 °C for at least 6 h, after which a multimembrane composite consisting of a sample absorbent membrane (GFB-R4), conjugate membrane (PT-R5), nitrocellulose membrane (CNPC-12μ), and absorbent membrane (AP045; all from Advanced Microdevices (Ambala Cantt, Haryana, India)) was collected. The lateral flow multimembrane composite was cut into test strips 3 mm wide using an Index Cutter-1 automatic guillotine (A-Point Technologies, Gibbstown, NJ, USA). The test strips were packed in laminated aluminum foil bags containing silica gel using an FR-900 continuous band sealer (Dingli Packing Machinery, Wenzhou, China).

### 2.8. Preparation of Enhancement Solution for Signal Amplification in LFIA

The enhancement solution consisted of equal volumes of solutions of hydroxylamine (0.5, 1, 2, 5, 10 mM in H_2_O) and HAuCl_4_ (1% in H_2_O). The solutions were mixed immediately before application to the test strip.

### 2.9. Scanning Electron Microscopy (SEM)

All SEM experiments were performed using a JSM-6510LV microscope (Jeol, Tokyo, Japan). After analysis in buffer, membranes were washed with water, dried, and coated with gold using an IB-3 coater (EIKO, Tokyo, Japan). Digital images were analyzed using the Image Tool program (UTHSCSA, San Antonio, TX, USA).

### 2.10. The Preparation of the Tuber Extract

The potato tubers without the peel were thoroughly homogenized in PBS with 0.05% Triton X-100 (PBST) (1:10 ratio by mass) in a porcelain mortar. Without further purification, the obtained extracts were used. The extracts of the healthy tubers were used for the dilution. To produce artificially contaminated samples, bacterial aliquots were added to healthy extracts. To study the assay recovery, we prepared 18 samples containing different amounts of *R. solanacearum* from 1 × 10^5^ to 10^8^ cells·mL^−1^ according to the linear range of the calibration curves for the assays with and without enhancement. Then, we compared the spiked concentration with the concentration found by the calibration curve. Each sample was measured three times.

### 2.11. Performance of Conventional LFIA and LFIA with Gold Enhancement

Conventional LFIA was carried out in PBST and extracts of potato tubers (sample/PBST ratio of 1:10 (w:w)). The test strip was dipped vertically into the analyzed sample and kept there for 10 min. After this, for LFIA with gold enhancement, we applied an enhancement solution (20 μL) to the wet test strips in the test zone area. These were kept in place for about 3 min and then washed with water.

The LFIA experiments were performed at least in triplicate. The visual LOD of the LFIA was defined as the *R. solanacearum* concentration when the test line appeared. For quantitative analysis, we scanned the test strips using a Cannon 9000F Mark II scanner (Canon, Tokyo, Japan) and analyzed the digital images using TotalLab TL120 (Nonlinear Dynamics, Newcastle upon Tyne, UK). The quantitative results are expressed as the mean of the data. The error bars on the calibration curves present the standard deviation.

### 2.12. Bacteria Cultivation and PCR Analysis

*R. solanacearum* was cultivated and confirmed by PCR analysis, as described in reference [41].

## 3. Results and Discussion

### 3.1. Scheme of the LFIA with Gold Enhancement

**Figure 1:**
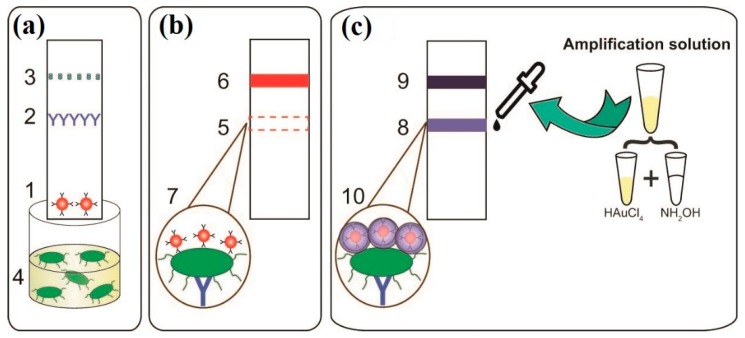
Lateral flow immunoassay (LFIA) scheme before and after signal amplification: (**a**) test strip dipped into a sample of potato tuber extract; (**b**) test strip after conventional LFIA, indicating a negative result because no color band exists in the test zone; (**c**) addition of amplification solution to the test strip and enlargement of the gold nanoparticle (GNP) size. The numbers in the figure indicate (1) GNP–antibody conjugate on the conjugate pad, (2) antibody specific to *R. solanacearum* immobilized in the test zone, (3) protein A immobilized on the control zone, (4) potato tuber extract containing *R. solanacearum*, (5) no visible color band in the test zone after analysis, (6) a clear red color band in the control zone, (7) formed immune complex (immobilized specific antibodies, *R. solanacearum*, GNP conjugate with specific antibody) after conventional LFIA, (8) test zone after signal amplification, (9) control zone after signal amplification, and (10) enlarged GNP particle size caused by the addition of amplification solution.

To carry out the LFIA, we used the sandwich LFIA format [44]. The sandwich format was based on the formation of triple immune complexes (immobilized specific antibodies, an antigen (*R. solanacearum*), a GNP conjugate with a specific antibody). An antibody specific to *R. solanacearum* was immobilized in the test zone, and protein A binding the antibodies on the surface of the GNP–antibody conjugate was immobilized in the control zone (Figure 1a).

When the test strip was dipped into the sample, the fluid migrated through the membrane via capillary forces. If a sample contains *R. solanacearum* when it passes the test zone, the *R. solanacearum* will interact with the antibodies conjugated with the GNPs, which is accompanied by the formation of an antigen–antibody complex. This complex is then bound in the test zone because of the interaction with immobilized specific antibodies. The uncaptured GNP–antibody conjugate then moves farther and is bound in the control zone, where it interacts with protein A. The appearance of a colored band in the control zone confirms the accuracy of the analysis and the diagnostic capabilities of the system. A negative result is shown by the absence of a colored band in the test zone, which indicates the lack of *R. solanacearum* in the sample or a concentration lower than the threshold level (Figure 1b). A positive result is indicated by the appearance of two bands (in both the test zone and control zone).

GNPs with larger size have higher coloration [45]. However, the application of large GNPs (about 100 nm) as a label in LFIA is limited by low stability and a low migration rate in the porous membrane. Kim et al. [46] showed that an increase of GNP size from 42.7 ± 0.8 nm up to 137.8 ± 0.4 nm results in a continuous decrease of the LOD of the hepatitis B surface antigen. Also, we showed earlier that the LOD of potato virus X for larger GNPs (about 52 nm) is higher due to steric hindrance [47].

To overcome the contradiction between the preference for larger nanoparticles for sensitive detection and the preference for smaller nanoparticles for better movement and the exclusion of nonspecific binding, we used initially small GNPs (about 20 nm) at the lateral flow stage of the assay and the following post-assay GNP growth. Small GNPs are stable, migrate well in the porous membrane, and form immune complexes in the test zone. The post-assay growth of GNPs allows for combining the advantages of small and large GNPs in LFIA.

Gold enlargement in the test zone of a lateral flow test strip is a special solution for LFIA amplification to diagnose potato brown rot in the absence of visible symptoms of infection. We conducted the gold enhancement using a mix of hydroxylamine and HAuCl_4_ solutions. The enhanced solution was dropped on the test zone, which started the reduction of tetrachloroaurate (III) ions by hydroxylamine on the GNP surface. If the test zone contains a small amount of triple immune complexes with the incorporation of the GNP conjugate in a concentration invisible to the eye, this can lead to the formation of a gold coating on the surface of the GNP conjugate. A color signal that was invisible because of a low concentration of *R. solanacearum* is amplified; thus, the test zone becomes a visible violet color (Figure 1c).

### 3.2. Characterization of the Bacterium, Synthesized Gold Nanoparticles, and Gold Nanoparticle–Antibody Conjugates

**Figure 2:**
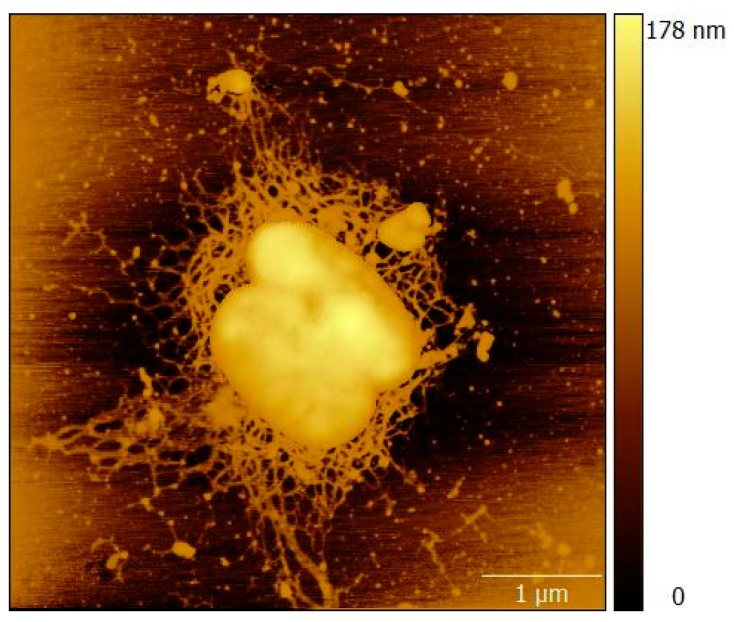
AFM image of *R. solanacearum*.

The bacterium used in the study was determined and confirmed as *R. solanacearum* by PCR. We used atomic force microscopy (AFM) measurements to confirm the integrity of *R. solanacearum* in a pure culture, which is important for developing sandwich LFIA. A typical AFM image of *R. solanacearum* is presented in Figure 2, which shows the rod-shaped cells, 0.5–1.5 µm in length, with a flagellum, corresponding to the typical morphology of *R. solanacearum* [48].

**Figure 3:**
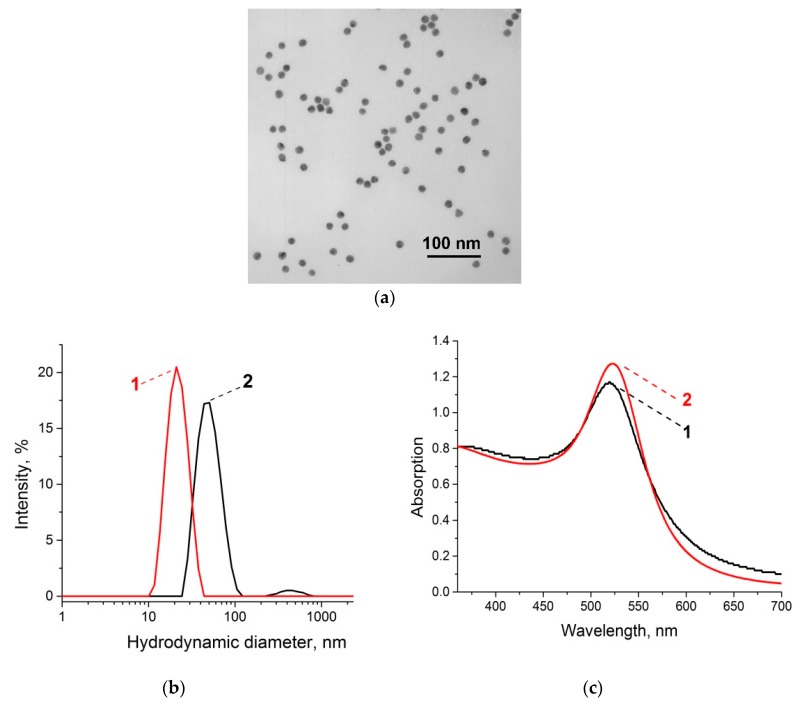
Characterization of the GNPs and GNP–antibody conjugate: (**a**) TEM image of GNPs; (**b**) DLS graphs of GNPs (1) and GNP–antibody conjugate (2); (**c**) spectra of GNPs (1) and the conjugate (2).

We synthesized the GNPs with an average diameter of 17.4 ± 1.0 nm and a form factor (i.e., the ratio of the maximum and minimum axes) of 1.1 ± 0.1 (data from transmission electron microscopy (TEM), Figure 3a). The homogeneity of the GNPs in the solution and the absence of aggregates was shown by dynamic light scattering (DLS) (Figure 3b), which also confirmed the dimensions of the nanoparticles (the average hydrodynamic diameter was 19.5 nm). Based on our previous experiences in the synthesis of GNP conjugates, we confirmed that spherical GNPs with diameters of 20–30 nm can provide high stability to the conjugate [33,34]. The absorption maximum of the synthesized GNP is about 520 nm (Figure 2c, curve 1). The results given above correspond to the criteria of a narrow GNP size distribution [22].

Based on the obtained GNPs, we synthesized a GNP–antibody conjugate. A flocculation curve was used to determine the minimum antibody concentration that prevents the aggregation of GNPs at high ionic strength (addition of NaCl up to 1% final concentration). The addition of salt results in a decrease of the electrostatic repulsion between GNPs due to changes in the electric double layer [49]. Antibodies adsorbed on the GNPs protect them from aggregation. We chose an antibody concentration equal to 12 µg·mL^−1^, at which no aggregation of GNPs was observed as recorded at a wavelength of 580 nm (see Figure S1 with the corresponding flocculation curve). The GNP–antibody conjugate was characterized by DLS and spectroscopy. After antibody immobilization, we observed the changes in the hydrodynamic diameter (an increase of up to 47.2 nm) (Figure 3b, curve 2) and the absorption maximum of the conjugate (an increase of up to 524 nm) (Figure 3c, curve 2), which confirmed the effective immobilization of the antibody on the GNP surface [47]. The synthesized conjugates were homogenous and show high reproducibility (see the narrow size distribution in Figure 3b and the adsorption peak in Figure 3c). The antigen-binding capacity of the conjugates was confirmed by the formation of the red bands on the nitrocellulose membrane with adsorbed *R. solanacearum*.

### 3.3. Optimization of the Enhancement Solution Composition

**Figure 4:**
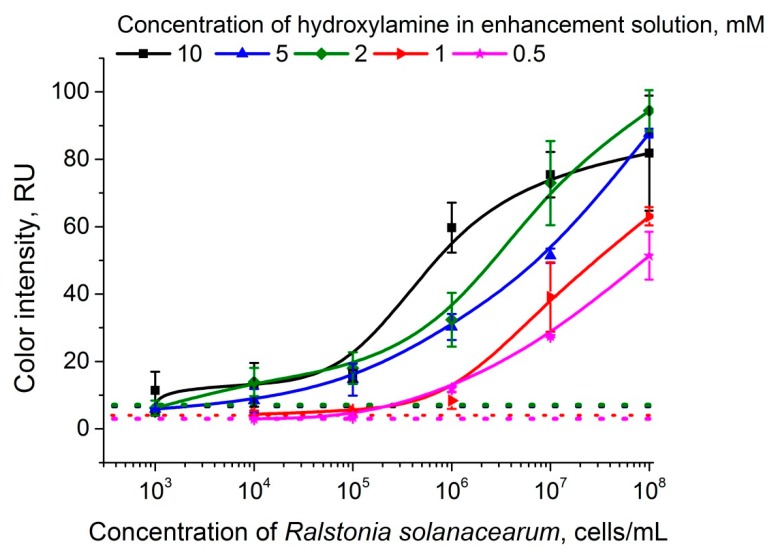
Calibration curves of *R.* *solanacearum* after enlargement with the enhancement solutions with different hydroxylamine concentrations. The dotted line corresponds to the LOD level.

The formation of methods for the practical task of diagnosing potato brown rot is associated with the need to detect the minimum possible concentrations of *R. solanacearum* in a sample and to increase the reliability of the analysis. We were guided by these two criteria in optimizing the composition of the enhancement solution. We used varied hydroxylamine concentrations (from 0.5 to 10 mM). A typical view of the test strips after lateral flow analysis with amplification at different hydroxylamine concentrations is shown in Table 1; the corresponding calibration dependences are shown in Figure 4. As shown in Figure 4, high hydroxylamine concentrations (from 2 to 10 mM) cause a strong color change from red to dark violet and significantly enhance the signal in the test zones. Hydroxylamine concentrations equal to 1 and 0.5 mM caused a slight increase in the signal in the test zones. However, nonspecific reactions were observed at a hydroxylamine concentration above 2 mM (see Figure 4). As a result, the LODs in the sequential concentration series from 0.5 to 10 mM were 3 × 10^5^, 1 × 10^5^, 2 × 10^4^, 6 × 10^4^, and 4 × 10^4^ cells·mL^−1^. Thus, the maximum reliability of the analysis and the minimum LOD were provided by a hydroxylamine concentration of 2 mM. This concentration was used in all further experiments.

### 3.4. Characterization of the Gold Enhancement on the Different Surfaces by AFM and SEM

**Figure 5:**
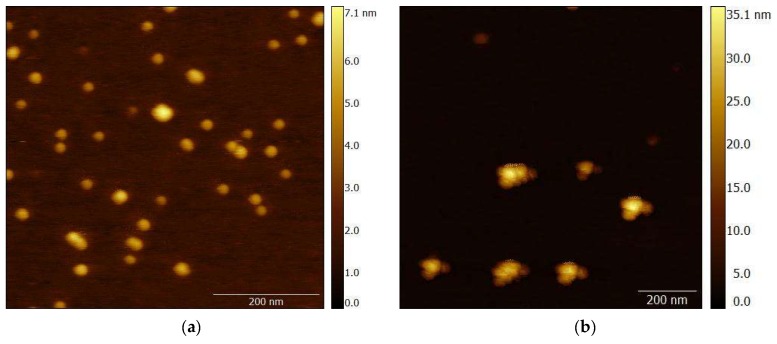

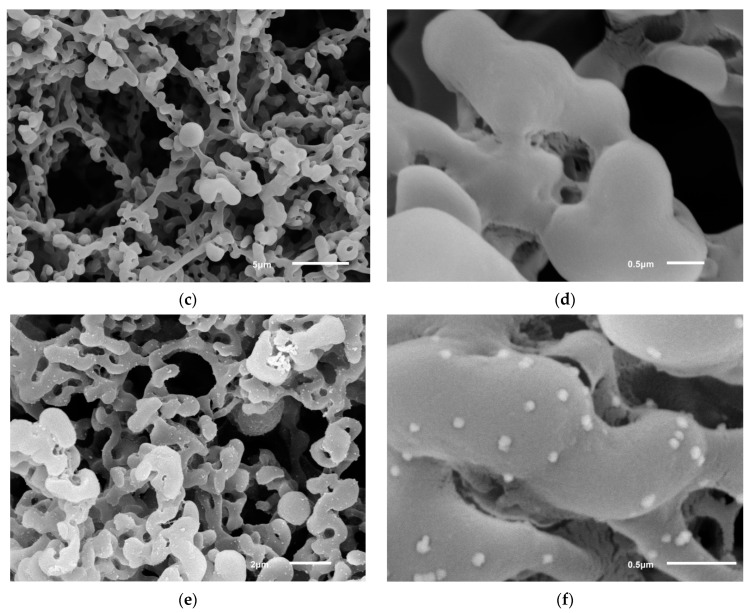
Gold enlargement on mica support and nitrocellulose membrane: (**a**) AFM images of initial GNPs; (**b**) GNPs after enlargement; (**c**,**e**) SEM images at different zooms of the initial GNPs; (**d**,**f**) GNPs after enlargement.

We characterized the gold enhancement caused by the reduction of tetrachloroaurate (III) ions by hydroxylamine on the GNP surface on the mica support and on the nitrocellulose membrane. The size increase of the GNPs was approximately up to 100 nm and was confirmed by AFM (Figure 5a,b) and SEM (Figure 5c–f) for both supports. The visualized enlarged GNPs had a narrow size distribution. Measurement of the surface texture of GNPs and GNPs after enlargement by AFM showed that the reduction of tetrachloroaurate (III) ions occurs evenly on the GNP surface. The roughness of the enlarged particles (RMS = 3.4 ± 0.6 nm, N = 15) increased significantly as compared with that of the initial GNPs (RMS = 0.3 ± 0.1 nm, N = 20). However, the reached roughness is still significantly lower than the diameter of the enlarged GNPs (up to 100 nm) and thus does not change the GNP shape significantly. Therefore, we can assume high reproducibility of the reaction, which is important for LFIA with gold enhancement.

### 3.5. LFIA in Buffer

**Figure 6:**
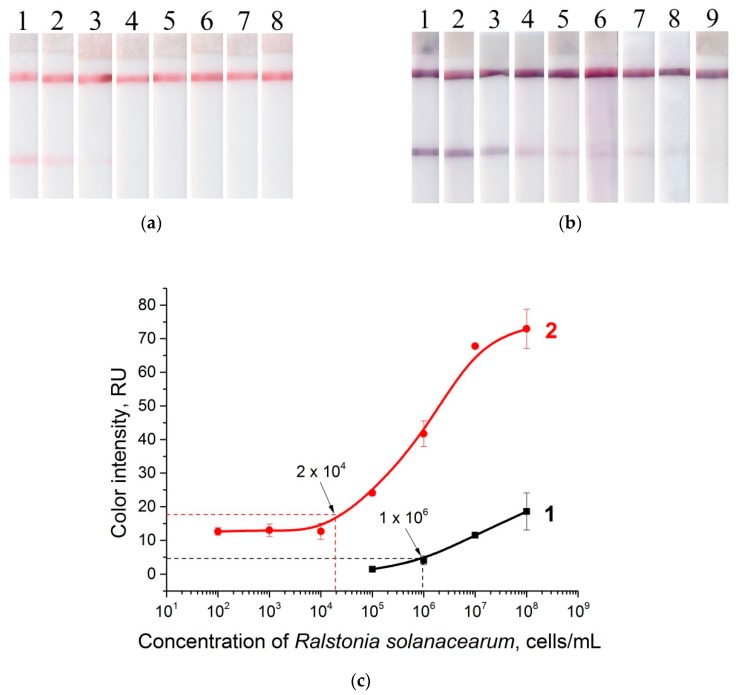
LFIA in buffer: (**a**) strips after testing and before enlargement; (**b**) strips after testing and enlargement. Strips #1–8 correspond to 10^8^, 10^7^, 10^6^, 10^5^, 10^4^, 10^3^, 10^2^, and 10^1^ cells·mL^−1^ of *R. solanacearum*, respectively; strip #9 corresponds to a negative control (0 cells·mL^−1^); (**c**) calibration curves of LFIA of *R. solanacearum* before (1) and after (2) enlargement.

Test strips for the detection of *R. solanacearum* were prepared in accordance with the sandwich scheme of LFIA (see Figure 1). LFIA with gold enhancement was carried out in the extraction buffer (PBST). The test strips before and after enhancement are shown in Figure 6a,b. The dependences of the color-staining intensity of the test zone before and after amplification are presented in Figure 6c. The gold enhancement reduces the LOD of *R. solanacearum* by 50 times, to as low as 2 × 10^4^ cells·mL^−1^. For the characteristics of the specificity of the test system, widespread potato pathogens (*Clavibacter michiganensis* subsp. *sepedonicus*, *Pectobacterium carotovorum*, *Dickeya* sp.), strains isolated from other *Solanaceae* plants (*C. michiganensis* subsp. *michiganensis*, *Artrobacter castelli*), and *Pseudomonas syringae* as a species from a close genus were used. The specificity of LFIA and LFIA with gold enhancement was confirmed; no cross-reactivity of the test strips was observed (see Supplementary Materials, Figure S2). No coloration in the test zone for LFIA of nonspecific bacteria was observed (Figure S2a) Gold enlargement also does not result in nonspecific signals (Figure S2b).

The developed approach based on gold enhancement does not complicate analyses and requires only 3 min. This time required for the developed enhancement is comparable with those for other gold enhancement methods—1 min [28], 3 min [50], 5 min [10], and 10 min [27,29]. The developed assay including the sample preparation and gold enlargement requires 15 min.

### 3.6. LFIA in Potato Tuber Extract

**Figure 7:**
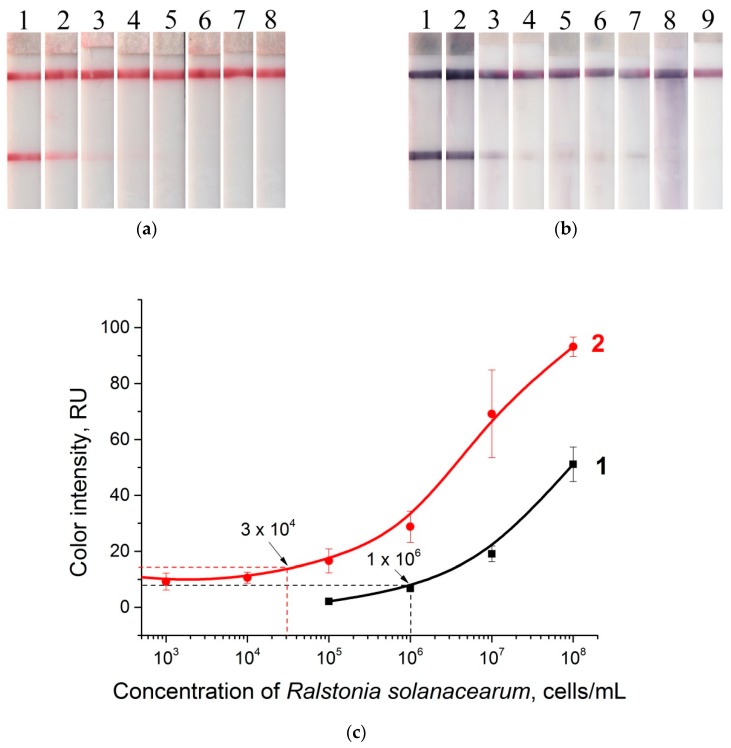
LFIA in potato tuber extract: (**a**) strips after testing and before enlargement; (**b**) strips after testing and enlargement. Strips #1–8 correspond to 10^8^, 10^7^, 10^6^, 10^5^, 10^4^, 10^3^, 10^2^, and 10^1^ cells·mL^−1^ of *R. solanacearum*, respectively, and strip #9 corresponds to a negative control (0 cells·mL^−1^); (**c**) calibration curves of LFIA of *R. solanacearum* before (1) and after (2) enlargement.

Signal amplification in potato tuber extracts was also carried out for a series of samples with a concentration of *R. solanacearum* varying from 10^1^ to 10^8^ cells·mL^−1^. The test strips before and after gold enhancement are shown in Figure 7a,b. The dependences of the color intensity of the test zone before and after amplification are presented in Figure 7c. The gold enhancement method decreased the LOD by 33 times, to as low as 3 × 10^4^ cells·mL^−1^. In comparison with buffer medium (see Figure 6), the amplification after analyzing the extracts leads to a small amount of nonspecific staining that affects the LOD. This is apparently due to the presence of the potato matrix components. For the gold enhancement carried out in the matrix, the effects of nonspecific coloration [27] or slow growth of GNPs [22] were described. The efficiency of the GNP enlargement approach based on reducing metallic salts (silver, gold, copper) depends on different matrixes and analytes. A detailed table with data about the LOD decrease for different assays is given in the Supplementary Materials (Table S1). In all these cases, the decrease in LOD is due to higher coloration of the formed enlarged particles in comparison with the initial seed particles. For bacteria, the LOD decrease observed was in the range from 8 (*Escherichia coli* O157: H7 in milk, [51]) to 100 times (for *Salmonella enteritidis* in milk [29]).

The result obtained by the gold enhancement method is lesser than the recorded values obtained earlier using silver enhancement (for which the detection limit of *R. solanacearum* is 200 cells·mL^−1^) [41]. However, the advantage of gold enhancement is a simpler and manually reproducible analysis protocol. This is an extremely important factor for the practical implementation of LFIA (for other gold enhancement methods, see Table S1). In addition, the LOD obtained in this work is lower than the LOD for commercially available test strips from Agdia, which is equal to 10^5^ CFU·mL^−1^ [52]. Methods based on PCR characterize a lower LOD (10^3^ for digital PCR [38], 10^2^ CFU·mL^−1^ for real-time PCR [39]). However, they require the use of expensive stationary equipment and reagents and take a long time; therefore, they cannot be used for the detection of *R. solanacearum* in non-laboratory conditions.

### 3.7. Recovery Experiment of LFIA with Gold Enhancement

**Figure 8:**
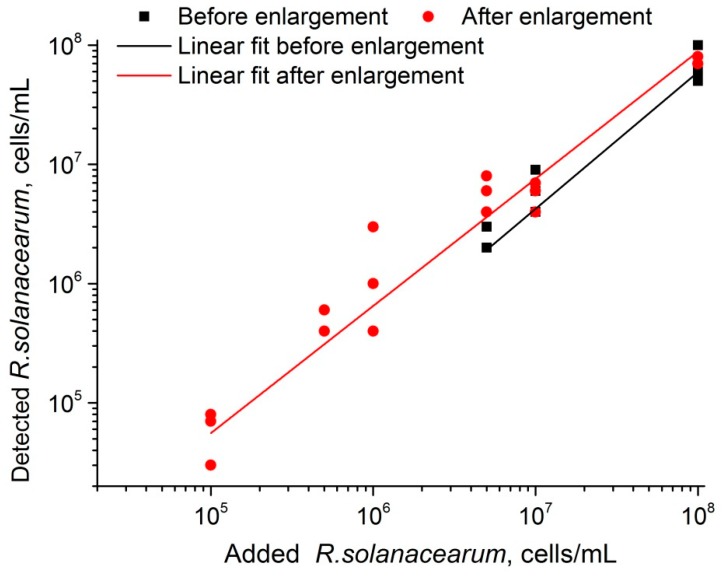
Correlation between added and detected *R.* *solanacearum* cells for LFIA before and after GNP enlargement.

LFIAs without and with enhancement were used to detect *R. solanacearum* in artificially contaminated samples (*n* = 18). As shown in Figure 8, both tests before and after enlargement showed good correlations (R^2^ = 0.99834 and 0.93943, correspondingly) with added values of *R. solanacearum.* Evidently, LFIA with amplification covers the concentration range at a much lower level (as low as 1 × 10^5^ cells·mL^−1^).

## 4. Conclusions

The developed approach does not complicate analysis and can be performed in a short period of time (about 3 min). At the same time, the achieved LOD of *R. solanacearum* (3 × 10^4^ cells·mL^–1^) allows the diagnosis of latent bacterial infection in potato tubers [53,54]. Thus, the developed approach is promising for the development of a lateral flow test system and subsequent introduction into diagnostic practice.

## Figures and Tables

**Table 1:** Optimization of the hydroxylamine concentration in the enhancement solution: test strips after gold enlargement with different hydroxylamine concentrations.

Concentration of hydroxylamine, mM	*R. solanacearum*, cells · mL^−1^						
	10^8^	10^7^	10^6^	10^5^	10^4^	10^3^	0 (control)
0.5	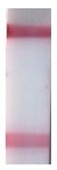	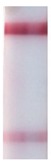	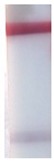				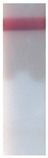
1	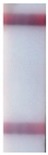	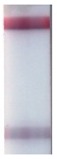	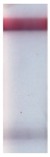	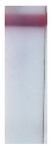			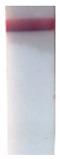
2	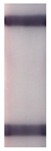	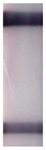	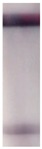	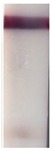	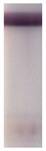		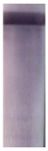
5	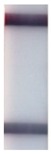	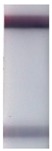	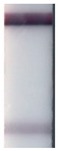	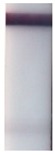	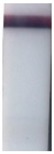		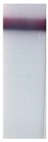
10	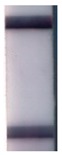	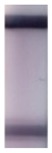	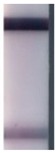	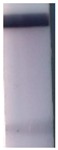	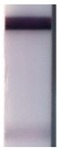	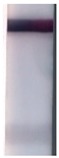	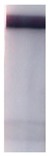

## Supplementary Materials

The following are available online at http://www.mdpi.com/1424-8220/19/1/153/s1, Figure S1: Flocculation curve for antibody to *Ralstonia solanacearum* and GNPs, Figure S2: LFIA of non-specific bacteria. (**a**) Test strips before enlargement. (**b**) Test strips after enlargement. 1-*Clavibacter michiganensis* subsp. *sepedonicus*, 2-*Pectobacterium carotovorum*, 3-*Dickeya* sp., 4-*Clavibacter michiganensis* subsp. *michiganensis*, 5-*Artrobacter castelli*, 6-*Pseudomonas syringae*, Table S1: Comparison of GNP enlargement based methods for highly sensitive assays.

## References

[1] Banerjee R., Jaiswal A. (2018). Recent advances in nanoparticle-based lateral flow immunoassay as a point-of-care diagnostic tool for infectious agents and diseases. Analyst.

[2] Nayak S., Blumenfeld N.R., Laksanasopin T., Sia S.K. (2017). Point-of-care diagnostics: Recent developments in a connected age. Anal. Chem..

[3] Lopez-Marzo A.M., Merkoci A. (2016). Paper-based sensors and assays: A success of the engineering design and the convergence of knowledge areas. Lab Chip.

[4] Dzantiev B.B., Byzova N.A., Urusov A.E., Zherdev A.V. (2014). Immunochromatographic methods in food analysis. TrAC-Trends Anal. Chem..

[5] Van Amerongen A., Veen J., Arends H.A., Koets M., Vashist S.K., Luong J.H.T. (2018). Chapter 7—Lateral flow immunoassays. Handbook of Immunoassay Technologies.

[6] Huang X., Aguilar Z.P., Xu H., Lai W., Xiong Y. (2016). Membrane-based lateral flow immunochromatographic strip with nanoparticles as reporters for detection: A review. Biosens. Bioelectron..

[7] Zherdev A.V., Dzantiev B.B. (2018). Ways to reach lower detection limits of lateral flow immunoassays. Rapid Test—Advances in Design, Format and Diagnostic Applications.

[8] Ye H., Xia X. (2018). Enhancing the sensitivity of colorimetric lateral flow assay (CLFA) through signal amplification techniques. J. Mater. Chem. B.

[9] Shan S., Lai W., Xiong Y., Wei H., Xu H. (2015). Novel strategies to enhance lateral flow immunoassay sensitivity for detecting foodborne pathogens. J. Agric. Food Chem..

[10] Dias J.T., Svedberg G., Nystrand M., Andersson-Svahn H., Gantelius J. (2017). Rapid signal enhancement method for nanoprobe-based biosensing. Sci. Rep..

[11] Wei H., Wang E. (2013). Nanomaterials with enzyme-like characteristics (nanozymes): Next-generation artificial enzymes. Chem. Soc. Rev..

[12] Lathwal S., Sikes H.D. (2016). Assessment of colorimetric amplification methods in a paper-based immunoassay for diagnosis of malaria. Lab Chip.

[13] Gao X., Xu L.-P., Wu T., Wen Y., Ma X., Zhang X. (2016). An enzyme-amplified lateral flow strip biosensor for visual detection of microRNA-224. Talanta.

[14] Gupta S., Huda S., Kilpatrick P.K., Velev O.D. (2007). Characterization and optimization of gold nanoparticle-based silver-enhanced immunoassays. Anal. Chem..

[15] Liu R., Zhang Y., Zhang S., Qiu W., Gao Y. (2014). Silver Enhancement of gold nanoparticles for biosensing: From qualitative to quantitative. Appl. Spectrosc. Rev..

[16] Yang W., Li X.-B., Liu G.-W., Zhang B.-B., Zhang Y., Kong T., Tang J.-J., Li D.-N., Wang Z. (2011). A colloidal gold probe-based silver enhancement immunochromatographic assay for the rapid detection of abrin-a. Biosens. Bioelectron..

[17] Yu Q., Li H., Li C., Zhang S., Shen J., Wang Z. (2015). Gold nanoparticles-based lateral flow immunoassay with silver staining for simultaneous detection of fumonisin B1 and deoxynivalenol. Food Control.

[18] Anfossi L., Di Nardo F., Giovannoli C., Passini C., Baggiani C. (2013). Increased sensitivity of lateral flow immunoassay for ochratoxin A through silver enhancement. Anal. Bioanal. Chem..

[19] Xing C., Kuang H., Hao C., Liu L., Wang L., Xu C. (2014). A silver enhanced and sensitive strip sensor for Cadmium detection. Food Agric. Immunol..

[20] Drygin Y.F., Blintsov A.N., Grigorenko V.G., Andreeva I.P., Osipov A.P., Varitzev Y.A., Uskov A.I., Kravchenko D.V., Atabekov J.G. (2012). Highly sensitive field test lateral flow immunodiagnostics of PVX infection. Appl. Microbiol. Biotechnol..

[21] Panferov V.G., Safenkova I.V., Byzova N.A., Varitsev Y.A., Zherdev A.V., Dzantiev B.B. (2018). Silver-enhanced lateral flow immunoassay for highly-sensitive detection of potato leafroll virus. Food Agric. Immunol..

[22] Byzova N.A., Zherdev A.V., Sveshnikov P.G., Sadykhov E.G., Dzantiev B.B. (2015). Development of an immunochromatographic test system for the detection of *Helicobacter pylori* antigens. Appl. Biochem. Microbiol..

[23] Liu C.C., Yeung C.Y., Chen P.H., Yeh M.K., Hou S.Y. (2013). Salmonella detection using 16S ribosomal DNA/RNA probe-gold nanoparticles and lateral flow immunoassay. Food Chem..

[24] Rodríguez M.O., Covián L.B., García A.C., Blanco-López M.C. (2016). Silver and gold enhancement methods for lateral flow immunoassays. Talanta.

[25] Habib A., Tabata M., Wu Y. (2005). Formation of gold nanoparticles by good’s buffers. Bull. Chem. Soc. Jpn..

[26] Engelbrekt C., Wagner M., Christiansen M.U.-B., Christensen H.E.M., Qian X., Ulstrup J., Zhao C., Zhang J. (2016). Side effect of good’s buffers on optical properties of gold nanoparticle solutions. ChemElectroChem.

[27] Lan M., Guo Y., Zhao Y., Liu Y., Gui W., Zhu G. (2016). Multi-residue detection of pesticides using a sensitive immunochip assay based on nanogold enhancement. Anal. Chim. Acta.

[28] Panferov V.G., Safenkova I.V., Zherdev A.V., Dzantiev B.B. (2018). Post-assay growth of gold nanoparticles as a tool for highly sensitive lateral flow immunoassay application to the detection of potato virus X. Microchim. Acta.

[29] Bu T., Huang Q., Yan L., Huang L., Zhang M., Yang Q., Yang B., Wang J., Zhang D. (2018). Ultra technically-simple and sensitive detection for *Salmonella enteritidis* by immunochromatographic assay based on gold growth. Food Control.

[30] Newman J.D.S., Blanchard G.J. (2006). Formation of gold nanoparticles using amine reducing agents. Langmuir.

[31] Li J., Zou M., Chen Y., Xue Q., Zhang F., Li B., Wang Y., Qi X., Yang Y. (2013). Gold immunochromatographic strips for enhanced detection of avian influenza and newcastle disease viruses. Anal. Chim. Acta.

[32] Uwamahoro F., Berlin A., Bucagu C., Bylund H., Yuen J. (2018). Potato bacterial wilt in Rwanda: Occurrence, risk factors, farmers’ knowledge and attitudes. Food Secur..

[33] Yuliar, Nion Y.A., Toyota K. (2015). Recent trends in control methods for bacterial wilt diseases caused by *Ralstonia solanacearum*. Microbes Environ..

[34] Mansfield J., Genin S., Magori S., Citovsky V., Sriariyanum M., Ronald P., Dow M., Verdier V., Beer S.V., Machado M.A. (2012). Top 10 plant pathogenic bacteria in molecular plant pathology. Mol. Plant Pathol..

[35] Elphinstone J.G., Allen C., Prior P., Hayward A.C. (2005). The current bacterial wilt situation: A global review. Bacterial Wilt: The Disease and the Ralstonia solanacearum Species Complex.

[36] Birch P.R.J., Bryan G., Fenton B., Gilroy E.M., Hein I., Jones J.T., Prashar A., Taylor M.A., Torrance L., Toth I.K. (2012). Crops that feed the world 8: Potato: Are the trends of increased global production sustainable?. Food Secur..

[37] Cellier G., Moreau A., Chabirand A., Hostachy B., Ailloud F., Prior P. (2015). A duplex PCR assay for the detection of *Ralstonia solanacearum* phylotype II strains in *Musa* spp.. PLoS ONE.

[38] Dreo T., Pirc M., Ramšak Ž., Pavšič J., Milavec M., Žel J., Gruden K. (2014). Optimising droplet digital PCR analysis approaches for detection and quantification of bacteria: A case study of fire blight and potato brown rot. Anal. Bioanal. Chem..

[39] Massart S., Nagy C., Jijakli M.H. (2014). Development of the simultaneous detection of *Ralstonia solanacearum* race 3 and *Clavibacter michiganensis* subsp. *sepedonicus* in potato tubers by a multiplex real-time PCR assay. Eur. J. Plant Pathol..

[40] Nikitin M.M., Statsyuk N.V., Frantsuzov P.A., Dzhavakhiya V.G., Golikov A.G. (2018). Matrix approach to the simultaneous detection of multiple potato pathogens by real-time PCR. J. Appl. Microbiol..

[41] Panferov V.G., Safenkova I.V., Varitsev Y.A., Drenova N.V., Kornev K.P., Zherdev A.V., Dzantiev B.B. (2016). Development of the sensitive lateral flow immunoassay with silver enhancement for the detection of *Ralstonia solanacearum* in potato tubers. Talanta.

[42] Frens G. (1973). Controlled nucleation for the regulation of the particle size in monodisperse gold suspensions. Nat. Phys. Sci..

[43] Byzova N.A., Safenkova I.V., Chirkov S.N., Zherdev A.V., Blintsov A.N., Dzantiev B.B., Atabekov I.G. (2009). Development of immunochromatographic test systems for express detection of plant viruses. Appl. Biochem. Microbiol..

[44] Bahadır E.B., Sezgintürk M.K. (2016). Lateral flow assays: Principles, designs and labels. TRAC Trends Anal. Chem..

[45] Khlebtsov N.G. (2008). Determination of size and concentration of gold nanoparticles from extinction spectra. Anal. Chem..

[46] Kim D.S., Kim Y.T., Hong S.B., Kim J., Huh N.S., Lee M.-K., Lee S.J., Kim B.I., Kim I.S., Huh Y.S. (2016). Development of lateral flow assay based on size-controlled gold nanoparticles for detection of hepatitis B surface antigen. Sensors.

[47] Safenkova I.V., Zherdev A.V., Dzantiev B.B. (2012). Factors influencing the detection limit of the lateral-flow sandwich immunoassay: A case study with potato virus X. Anal. Bioanal. Chem..

[48] Tans-Kersten J., Huang H., Allen C. (2001). *Ralstonia solanacearum* needs motility for invasive virulence on tomato. J. Bacteriol..

[49] Adamson A.W. (1990). Physical Chemistry of Surfaces.

[50] Shin J.H., Hong J., Go H., Park J., Kong M., Ryu S., Kim K.-P., Roh E., Park J.-K. (2018). Multiplexed detection of foodborne pathogens from contaminated lettuces using a handheld multistep lateral flow assay device. J. Agric. Food Chem..

[51] Wang J.-Y., Chen M.-H., Sheng Z.-C., Liu D.-F., Wu S.-S., Lai W.-H. (2015). Development of colloidal gold immunochromatographic signal-amplifying system for ultrasensitive detection of *Escherichia coli* O157:H7 in milk. RSC Adv..

[52] Li X., Nie J., Hammill D.L., Smith D., Xu H., De Boer S.H. (2014). A comprehensive comparison of assays for detection and identification of *Ralstonia solanacearum* race 3 biovar 2. J. Appl. Microbiol..

[53] Hayward A.C. (1974). Latent infections by bacteria. Annu. Rev. Phytopathol..

[54] Lund B.M., Kelman A. (1977). Determination of the potential for development of bacterial soft rot of potatoes. Am. Potato J..

