# Supplementary material for: Enlargement of Gold Nanoparticles for Sensitive Immunochromatographic Diagnostics of Potato Brown Rot
Source: Sensors (Basel). 2019 Jan 4;19(1):153. doi: 10.3390/s19010153 (PMC6338966; doi:10.3390/s19010153)
Supplement: Supplementary file 1 [file sensors-19-00153-s001.pdf]

sensors

MDPI

Electronic Supplementary Material

Enlargement of Gold Nanoparticles for Sensitive Immunochromatographic Diagnostics of Potato Brown Rot

Shyatesa C. Razo [1,2], Natalia A. Panferova [1], Vasily G. Panferov [1], Irina V. Safenkova [1], Natalia V. Drenova [3], Yuri A. Varitsev [4], Anatoly V. Zherdev [1], Elena N. Pakina [2] and Boris B. Dzantiev [1,*]

[1] A.N. Bach Institute of Biochemistry, Research Centre of Biotechnology of the Russian Academy of Sciences, Leninsky Prospect 33, 119071 Moscow, Russia; kish218@gmail.com (S.C.R.); nemchenko.na@yandex.ru (N.A.P.); panferov-vg@mail.ru (V.G.P.); saf-iri@yandex.ru (I.V.S.); zherdev@inbi.ras.ru (A.V.Z.)

[2] Agricultural-Technological Institute, RUDN University, Miklukho-Maklaya Street 8/2, 117198 Moscow, Russia; e-pakina@yandex.ru

[3] All-Russian Plant Quarantine Centre, Pogranichnaya Street, 32, Bykovo-2, 140150 Moscow, Russia; drenova@mail.ru

[4] A.G. Lorch All-Russian Potato Research Institute, Lorch Street 23, Kraskovo, 140051 Moscow, Russia; varyuriy@yandex.ru

* Correspondence: dzantiev@inbi.ras.ru; Tel.: +7-495-954-3142; Fax: +7-495-954-2804

Received: 23 November 2018; Accepted: 28 December 2018; Published: date

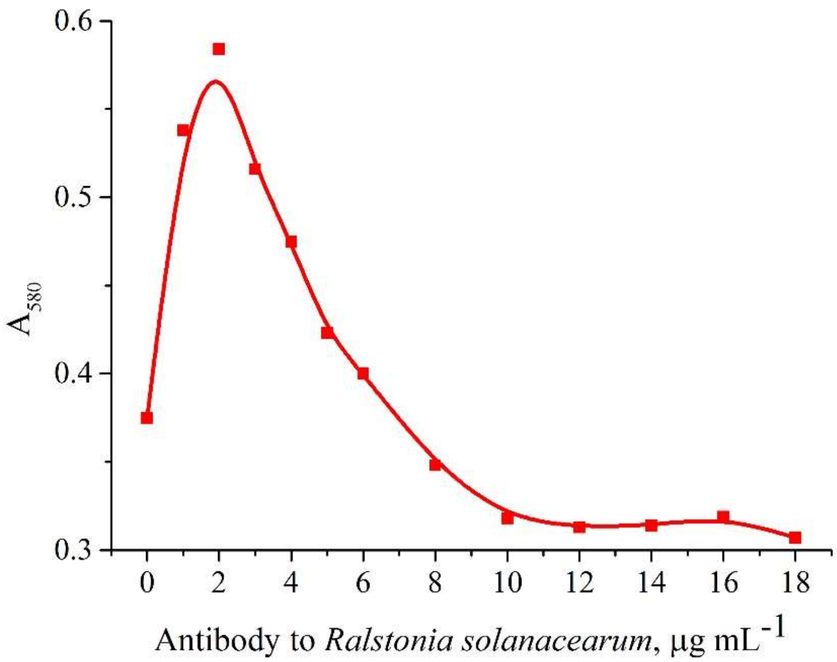

Figure S1. Flocculation curve for antibody to *Ralstonia solanacearum* and GNPs.

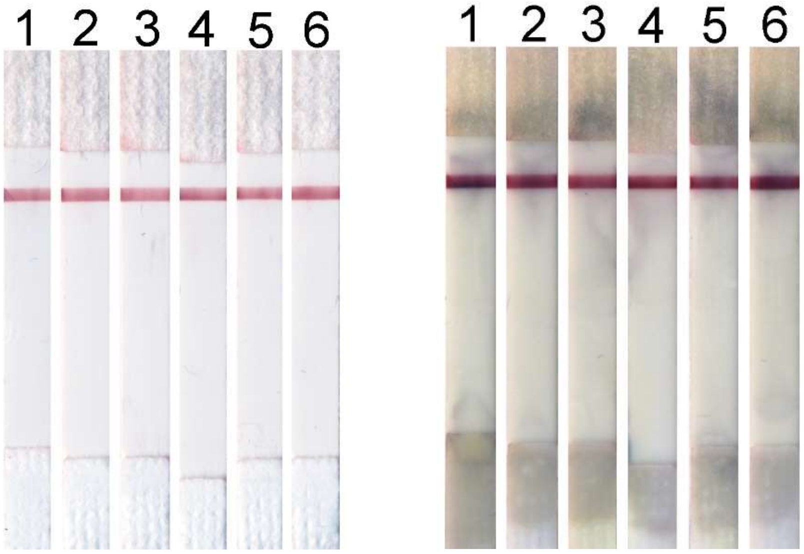

(**a**) (**b**)

Figure S2. LFIA of non-specific bacteria. (**a**) Test strips before enlargement. (**b**) Test strips after enlargement. 1–*Clavibacter michiganensis* subsp. *sepedonicus*, 2–*Pectobacterium carotovorum*, 3–*Dickeya* sp., 4–*Clavibacter michiganensis* subsp. *michiganensis*, 5–*Artrobacter castelli*, 6–*Pseudomonas syringae*.

Table S1. Comparison of GNP enlargement based methods for highly sensitive assays.

Assay	Analyte	Enlargement Strategy	LOD Decrease	Reference
LFIA	Deoxynivalenol<br>fumonisin B1	Silver enhancement	2-fold<br>2.5-fold	[1]
LFIA	Prostate-specific antigen	Silver enhancement	3-fold	[2]
LFIA	Cadmium	Silver enhancement	4-fold	[3]
LFIA	*Escherichia coli* O157:H7	Gold enhancement	8-fold	[4]
Paper immunoassay	*E.* O157:H7<br>*Salmonella typhimurium*	Gold enhancement	10-fold	[5]
LFIA	Ochratoxin A	Silver enhancement	10-fold	[6]
LFIA	*Helicobacter pylori* antigens	Silver enhancement	10-fold	[7]
LFIA	Potato leafroll virus	Silver enhancement	15-fold	[8]
Dot-blot immunoassay	*Mycobacterium tuberculosis* antigen	Silver enhancement<br>Copper enhancement	4-fold<br>17-fold	[9]
LFIA	Abrin-a	Silver enhancement	100-fold	[10]
LFIA	Avian influenza virus<br>Newcastle disease virus	Gold enhancement	100-fold	[11]
Flow through immunoassay	Protein G	Gold enhancement	100-fold	[12]
LFIA	*Salmonella enteridis*	Gold enhancement	100-fold	[13]
3D paper based assay	Human norovirus	Gold enhancement	100-fold	[14]
LFIA	Potato virus X	Gold enhancement	240-fold	[15]

References

1. Yu, Q.; Li, H.; Li, C.; Zhang, S.; Shen, J.; Wang, Z. Gold nanoparticles-based lateral flow immunoassay with silver staining for simultaneous detection of fumonisin B1 and deoxynivalenol. *Food Control* **2015**, *54*, 347–352, doi:10.1016/j.foodcont.2015.02.019.
2. Rodríguez, M.O.; Covián, L.B.; García, A.C.; Blanco-López, M.C. Silver and gold enhancement methods for lateral flow immunoassays. *Talanta* **2016**, *148*, 272–278, doi:10.1016/j.talanta.2015.10.068.
3. Xing, C.; Kuang, H.; Hao, C.; Liu, L.; Wang, L.; Xu, C. A silver enhanced and sensitive strip sensor for Cadmium detection. *Food Agr. Immunol.* **2014**, *25*, 287–300, doi:10.1080/09540105.2013.781140.
4. Wang, J.-Y.; Chen, M.-H.; Sheng, Z.-C.; Liu, D.-F.; Wu, S.-S.; Lai, W.-H. Development of colloidal gold immunochromatographic signal-amplifying system for ultrasensitive detection of Escherichia coli O157:H7 in milk. *RSC Adv.* **2015**, *5*, 62300–62305, doi: 10.1039/C5RA13279G.
5. Park, J.; Shin, J.H.; Park, J.-K. Pressed paper-based dipstick for detection of foodborne pathogens with multistep reactions. *Anal. Chem.* **2016**, *88*, 3781–3788, doi: 10.1021/acs.analchem.5b04743.
6. Anfossi, L.; Di Nardo, F.; Giovannoli, C.; Passini, C.; Baggiani, C. Increased sensitivity of lateral flow immunoassay for ochratoxin A through silver enhancement. *Anal. Bioanal. Chem.* **2013**, *405*, 9859–9867, doi:10.1007/s00216-013-7428-6.
7. Byzova, N.A.; Zherdev, A.V.; Sveshnikov, P.G.; Sadykhov, E.G.; Dzantiev, B.B. Development of an immunochromatographic test system for the detection of Helicobacter pylori antigens. *Appl. Biochem. Microbiol.* **2015**, *51*, 608–617, doi:10.1134/s000368381505004x.
8. Panferov, V.G.; Safenkova, I.V.; Byzova, N.A.; Varitsev, Y.A.; Zherdev, A.V.; Dzantiev, B.B. Silver-enhanced lateral flow immunoassay for highly-sensitive detection of potato leafroll virus. *Food Agr. Immunol.* **2018**, *29*, 445–457, doi:10.1080/09540105.2017.1401044.
9. Phan, L.M.T.; Rafique, R.; Baek, S.H.; Nguyen, T.P.; Park, K.Y.; Kim, E.B.; Kim, J.G.; Park, J.P.; Kailasa, S.K.; Kim, H.-J.; et al. Gold-copper nanoshell dot-blot immunoassay for naked-eye sensitive detection of tuberculosis specific CFP-10 antigen. *Biosens. Bioelectron.* **2018**, *121*, 111–117, doi: 10.1016/j.bios.2018.08.068.
10. Yang, W.; Li, X.-b.; Liu, G.-w.; Zhang, B.-b.; Zhang, Y.; Kong, T.; Tang, J.-j.; Li, D.-n.; Wang, Z. A colloidal gold probe-based silver enhancement immunochromatographic assay for the rapid detection of abrin-a. *Biosens. Bioelectron.* **2011**, *26*, 3710–3713, doi:10.1016/j.bios.2011.02.016.
11. Li, J.; Zou, M.; Chen, Y.; Xue, Q.; Zhang, F.; Li, B.; Wang, Y.; Qi, X.; Yang, Y. Gold immunochromatographic strips for enhanced detection of avian influenza and newcastle disease viruses. *Anal. Chim. Acta* **2013**, *782*, 54–58, doi: 10.1016/j.aca.2013.04.022.
12. Dias, J.T.; Svedberg, G.; Nystrand, M.; Andersson-Svahn, H.; Gantelius, J. Rapid signal enhancement method for nanoprobe-based biosensing. *Sci. Rep.* **2017**, *7*, 6837, doi:10.1038/s41598-017-07030-0.
13. Bu, T.; Huang, Q.; Yan, L.; Huang, L.; Zhang, M.; Yang, Q.; Yang, B.; Wang, J.; Zhang, D. Ultra technically-simple and sensitive detection for Salmonella enteritidis by immunochromatographic assay based on gold growth. *Food Control* **2018**, *84*, 536–543, doi: 10.1016/j.foodcont.2017.08.036.
14. Han, K.N.; Choi, J.-S.; Kwon, J. Three-dimensional paper-based slip device for one-step point-of-care testing. *Sci. Rep.* **2016**, *6*, 25710–25710, doi: 10.1038/srep25710.
15. Panferov, V.G.; Safenkova, I.V.; Zherdev, A.V.; Dzantiev, B.B. Post-assay growth of gold nanoparticles as a tool for highly sensitive lateral flow immunoassay application to the detection of potato virus X. *Microchim. Acta* **2018**, *185*, 506, doi:10.1007/s00604-018-3052-7.

© 2018 by the authors. Submitted for possible open access publication under the terms and conditions of the Creative Commons Attribution (CC BY) license (http://creativecommons.org/licenses/by/4.0/).
